# Experiences of risk in Australian hotel quarantine: a qualitative study

**DOI:** 10.1186/s12889-022-13339-x

**Published:** 2022-05-12

**Authors:** Bridget Haire, Gwendolyn L. Gilbert, John M. Kaldor, David Hendrickx, Angus Dawson, Jane H. Williams

**Affiliations:** 1 Kirby Institute, UNSW, Level 6, Wallace Wurth Building, Sydney, NSW 2052 Australia; 2 Australian Human Rights Institute, University of New South Wales, Sydney, Australia; 3 Sydney Health Ethics, University of Sydney Institute for Infectious Diseases, University of Sydney, Level 1, Edward Ford Building, A27, Sydney, NSW 2006 Australia; 4 Wesfarmers Centre for Vaccines and Infectious diseases, Telethon Kids Institute, Perth Children’s Hospital, 15 Hospital Ave., Nedlands, WA 6009 Australia; 5 Faculty of Health and Medical Sciences, University of Western Australia, Perth, Australia

**Keywords:** COVID-19, Quarantine, Qualitative research, Risk management, Public health policy

## Abstract

**Background:**

In response to the threat of COVID-19 infection, Australia mandated a 14 day quarantine period in a designated facility for all travellers returning from overseas from late March 2020. These facilities were usually hotels, or hotel-like serviced apartments, and also included a repurposed former mining village in the Northern Territory. This paper aimed to investigate the experiences of risk of people quarantined in designated supervised facilities in Australia, which has not been systematically explored before.

**Methods:**

In this qualitative study semi-structured interviews were conducted with 58 participants quarantined between March 2020 and January 2021. Participants were returned Australian citizens and residents who were required to undergo mandatory supervised quarantine for COVID-19. Interviews were conducted using video teleconferencing (via Zoom), transcribed and coded, then analysed thematically.

**Results:**

While participants generally supported the concept of quarantine to protect the Australian public, they were critical of elements of it where they felt exposed to risk (COVID-related or not). They also described instances where infection control within the system seemed inadequate. For some, particularly those quarantined with small children, they reported that the facilities were inadequate or inappropriate for health and wellbeing. Using thematic analysis, three major themes were identified that related to problems in the existing system: perception of being subjected to high risk through lax standards of COVID protection in the quarantine process; risks to the community identified in quarantine; and risk in non-hotel managed quarantine facilities.

**Conclusions:**

There are systemic issues with infection control in hotel quarantine, which can be further undermined by individual non-compliance. Risks to safety for those in quarantine can be reduced, both in terms of infection control within hotel quarantine and, in the case of the Northern Territory facility, timely in-person medical care as needed for non-COVID conditions. Systems of infection control need ongoing review to ensure that people entering quarantine are protected from known risks of infection at every stage. Medical services in quarantine facilities should be examined to ensure timely and appropriate non-COVID medical services are available.

## Background

Australia was among the first countries to implement hotel quarantine, commencing on 28 March 2020. At the time of writing, all incoming international travellers (except those from New Zealand) must quarantine for 14 days in a designated hotel or hotel-like serviced apartment or at Howard Springs (a former mining village in the Northern Territory repurposed as a quarantine facility). The deployment of hotels for quarantine purposes offers several advantages. From a security perspective it is easier to ensure that people do not break quarantine than if they were allowed home [1]. In addition, it provides income for facilities that would otherwise be largely vacant, due to border restrictions and lockdowns [2]. The disadvantage is that hotels are designed predominantly as short-term sleeping quarters, not as self-contained infection-secure living spaces for longer periods.

Australia is one of about 12 countries worldwide to implement the hotel-based system [3], which, in addition to accommodating incoming international travellers, entails transporting them to government approved hotel facilities for the quarantine period (or a longer, should they test positive). Hotels are allocated on the day of arrival. Quarantine was initially funded by government, but from mid-July 2020, partial user-payment was introduced [4]. The fee is fixed regardless of the hotel, but varies across jurisdictions [5–10]. Quarantine conditions and rules differ across states and territories, and also among hotels. For example, some allow fresh air or exercise breaks and unlimited deliveries, others not.

From its inception, issues relating to hotel quarantine have featured in media reports, and one study has investigated rates of psychological distress in hotel quarantine in South Australia in April 2020 [11]. To date however there has been no comprehensive qualitative research exploring the experience of people in Australia undergoing this novel form of quarantine.

## Methods

This was a qualitative study that collected data in the form of semi structured interviews. Participants were recruited through advertisements posted on social media sites, including hotel quarantine Facebook groups, the websites and social media accounts of the authors’ affiliated organisations, and through word-of-mouth. We conducted 73 semi-structured interviews. Participants included 58 who were in or had experienced quarantine in a designated quarantine facility, and 15 who had been in mandatory home quarantine before the end of March 2020. This analysis is focused on the 58 participants in designated facilities only.

### Participant selection

Recruitment comprised three distinct phases, with each phase responding either to a change in policy regarding hotel quarantine or to specific media reports of emerging issues.

In phase 1 we interviewed 30 people, of whom half had been in mandated home quarantine and half in hotel quarantine. This phase began just as hotel quarantine was introduced in Australia and when it was not clear how long it would be in place. The aim of interviewing the two sets of participants was to consider the differences between the two different forms of quarantine in terms both of risk reduction to the community and acceptability for those quarantined. By mid-2020 it was clear that hotel quarantine would remain Australia’s policy, and phase 2 of recruitment was triggered by media reports of Melbourne’s “second wave” of COVID-19 being linked to ‘leaks’ from two specific quarantine hotel. Accordingly, in phase 2 we interviewed 11 individuals from the two hotels identified as sources of community transmission in Melbourne with the aim of understanding whether there were identifiable differences in the experience of people at these hotels that might explain why the leaks occurred [12]. The interview schedule was modified to include questions about participants’ perceptions of risk.

The catalyst for phase 3 was the introduction of payment for hotel quarantine. In this phase we interviewed 26 people who were or had been quarantined in hotels, and six at a former mining camp, repurposed as a quarantine facility, in Howard Springs in the Northern Territory. The interview schedule was again modified, with questions about the impact of payment added, and the risk question adjusted to ask about the process of getting to hotels, as this had become a point of focus in media reports [13].

Recruitment ceased when we considered that we had a sample that was sufficiently large and diverse to ensure that we had a thorough in-depth understanding of key issues occurring in quarantine at that timepoint. Given the unique circumstances of each individual and the method of recruitment, we did not think it possible to aim to reach data saturation [14]. This approach is consistent with the reflexive thematic analysis methods used in this study.

### Data collection

All interviews were conducted online using the video conferencing platform Zoom by authors BH and JW, with informed consent obtained verbally. Several participants opted to be interviewed with a partner with whom they had been or were currently quarantining. In these cases, both provided informed consent.

Interviews lasted between 35 and 95 minutes, and most took an hour. Most interviews were conducted after participants had finished quarantine, but 12 were conducted while they were still quarantined, towards the end of their stay. We did not observe any difference between people interviewed during or after their quarantine. Eight people reported being quarantined more than once. Issues about privacy were discussed in detail with participants as part of the consenting process, and any participant who had concerns was invited to have transcripts returned for checking/redaction as required. Two participants took up this option. Interviews were recorded and transcribed by a professional transcriber.

### Research team

Both interviewers were PhD qualified cis-gender women trained in empirical bioethics who are experienced in conducting interviews on sensitive topics. However, prior to this study neither had conducted interviews by video conferencing. The two interviewers were based at different institutions. Two participants in the study were known to the interviewers through work connections, and in both cases the interview was assigned to avoid interviewing a colleague.

### Data analysis

The methodological approach of the study was empirical bioethics utilising reflexive thematic analysis [15, 16]. Transcripts were coded first using a descriptive framework drawn from the research questions, and then inductively to capture concepts. For example, the descriptive label ‘bus driver no mask’ was coded as ‘risk’, and subsequently formed part of the theme, ‘Feeling at risk in the quarantine process’. Both interviewers coded all interviews independently. Coding structures and the development of themes were explored through memos and discussion with the research team. BH and JW met regularly to discuss similarities and differences in interviews and to tease out themes and analysis. N-Vivo 12 was the software used the manage the data. Where quotes from participants are used, the state and date refer to the quarantine location and time. The study had ethics approval from the University of New South Wales Human Research Ethics Committee.

## Results

We report here on three main themes from 58 participants in designated quarantine facilities (see Table 1): Perception of being subjected to high risk through lax standards of COVID protection; identifying risk to the community through ‘holes’ in the process; and risk in non-hotel managed quarantine facilities.

**Table 1:** Summary of participant demographics, location and phase of data collection

Participant summary^a^		
**Gender**	**/58**	%
Male	19	33
Female	39	67
**Age**	**/58**	%
19–29	12	21
30–39	17	29
40–49	10	17
50–59	3	5
60–69	13	22
70–75	3	5
**Quarantine group**	**/59**^b^	%
Alone	29	49
With another adult	20	34
With partner and children	7	12
With children	3	5
**Location**	**/59**^a^	%
New South Wales	28	48
Victoria	13	22
Western Australia	2	3
Northern Territory^c^	6	10
South Australia	3	5
Queensland	7	12

^a^15 participants in mandatory home quarantine are not included in this table nor in this analysis

^b^Totals 59 because one participant had quarantined twice under different circumstances

^c^NT participants were quarantined in a repurposed designated quarantine centre, not a hotel

Phase 1: Quarantined between March and June 2020

Phase 2: Quarantined between March and July 2020 at two hotels in Melbourne

Phase 3: Quarantined between September 2020 and January 2021

### Perception of being subjected to high risk through lax standards of COVID protection

For many participants returning from high-risk regions where mask wearing had become normalised, the lack of consistent mask wearing in airports was very concerning. These participants reported feeling more at risk when travelling back to Australia than they had felt during the pandemic up to that point, and felt powerless to reduce this risk because they couldn’t control what was happening around them.

Many phase 3 participants reported that best practice risk reduction was not adhered to during the process of getting from the aeroplane to the hotel. Participants noted that while medical personnel usually wore personal protective equipment (PPE), other staff (including Australian Defence Force personnel, police and airline staff) often did not wear masks, were not adequately distanced from each other and/or the returning passengers, and left passengers waiting on buses together for extended periods without physical distancing or ventilation.

The thing that shocked me the most was that the bus driver was not wearing a mask for the entire time. … that was the most unsafe that I felt, was on the bus, that there was no social distancing, there was no point at any time where were told to keep our masks on. There [were] no windows, there was no ventilation. We were sat on the bus on the street for probably close to an hour*.*

Jade, mid-October 2020, phase 3, Sydney, NSW

One phase 3 participant also noted that traveller processing points both in airports and in the hotel were not located in well-ventilated areas, posing a potential risk from incoming travellers, to each other, and to the staff doing the processing.

There were so many other, better places there could’ve been that would’ve kept a better throughput of air for the purposes of ventilation and not had people kind of forced into close proximity.

Maeve, December 2020, phase 3, Sydney, NSW

Some participants in both phases 2 and 3 perceived the hotel accommodation itself as posing a high risk for acquiring SARS-CoV-2, and were vigilant about minimising it, including thorough hand hygiene after unwrapping each meal delivery, and cleaning and disinfecting rooms upon arrival.

Whenever I open the door and get my meals, I had always been washing my hands afterwards and, when I put rubbish in front of the door, I would come back and wash my hands again. My hands got really dry.

Hannah, June-July 2020, phase 1, Melbourne, VIC

Many phase 2 participants reported that their hotel rooms were very dirty and they felt that they were at risk of harm from non-COVID illness. Accounts of dirtiness included thick dust, mould and dirt in bathrooms, stained bedlinen, bits of food and used PPE under beds and floors too dirty to lie on for exercise.

There was faeces in the toilet … And there was urine all around the seat of the toilet. So, I told the hotel staff that, but it took four days to get a toilet brush and stuff to clean it. Which I, I, I was, I was absolutely disgusted, and I couldn’t even clean it*.*

Terri, April 2020, phase 2, Melbourne, VIC

In all three phases, there were participants with non-COVID illnesses and injuries who did not feel that they received adequate timely medical care. Specific issues included an inadequate response to severe pain caused by gout (reported by Beng, April 2020, phase 1, Brisbane, QLD); lack of basic first aid devices such as ice packs (reported by Isobel, June–July 2020, phase 2 Melbourne, VIC); diet related stomach problems that were not catered for (Sergio, April 2020, phase 1, Sydney, NSW).

Several participants reflected that this made them feel that the quarantine process did not take adequate care of people being quarantined, but was focused only on protecting the community outside.

You are treated like a leper, and it is a horrible, horrible experience. So the humanity side for me, is absolutely missing when you go into a quarantine hotel.

Martina, September 2020, phase 3, Sydney, NSW (Martina was also quarantined later in Howard Springs)

Participants also identified flaws in infection control instructions they received – such as being told to change their masks and then sanitise hands, rather than removing the old mask and sanitising, before donning the fresh one.

I think it should be removed the old mask, and then disinfect your hand, then put on the new mask. Because when you touch the old mask there might be the germs on it*.*

Charlie, January 2021, phase 3, Gold Coast, QLD

Several participants who were quarantined at the two Melbourne hotels associated with the second wave in Victoria noted that the security services appeared unprofessional and that this potentially contributed to risk. Several participants, whose rooms were positioned so that they could see the security guards stationed at either end of the corridor, reported seeing guards sleeping or on their phones. Another participant quarantined at the same time, however, suggested that security guards had been scapegoated for the infection control breach when there were other factors at play, such as, for example, inconsistent use of masks and gloves by all staff at this hotel.

I was horrified that they decided to attack the lowest common denominator in that outbreak, because there was so much wrong with [the hotel] that was not about security guards*.*

Jean, April 2020, phase 2, Melbourne, VIC

This theme highlights that from the perspective of people entering hotel quarantine, there were breaches in infection control that made participants feel vulnerable to acquiring SARS-CoV-2.

### Risks to the community identified in quarantine

We explored risks to the community identified in participants’ accounts of their experiences. Some participants were quarantined as early as 28 March 2020 (the day that the program began) and others as late as January 2021. During this period, there were changes to the implementation of the program that addressed some of the risk factors that participants identified, as noted in the following accounts.

Participants who underwent hotel quarantine early in the program were offered testing only if they reported any COVID-like symptoms. Several participants in phase 1 reported that although they had a sore throat, they attributed it to the constant air conditioning, did not report the symptom and were not tested (e.g. Margot, March–April 2020, phase 1, Sydney, NSW). Others agreed that they would not have reported symptoms due to the risk of extended quarantine.

But like, honestly, if I did have symptoms, I wouldn’t have told them because it would have meant I probably would have had to stay longer*.*

Hazel, April-May 2020, phase 1, Sydney, NSW

Our phase 2 sample included nine people who had been on a cruise ship, on which more than half the passengers ultimately tested positive to COVID-19. When ensconced in hotel quarantine in Melbourne, these participants reported that testing was still optional in April 2020, even when their previous COVID test had been positive. Participants reported being given the option of testing again – but having to stay another 14 days if positive – or not testing, and being released on time. This means that a positive person could easily have been discharged.

If we tested positive again, at the beginning they were talking about us then having to start our fourteen days from the positive test… but if we refused testing, that was ok, we’d go home on the same day that we were supposed to*.*

Jean, April 2020, phase 2, Melbourne, VIC

Some people travelling together from the cruise ship had discordant respiratory PCR results, but were nevertheless accommodated together in the hotel for several days, before there was a suggestion of separating PCR positive and negative people (which was resisted by participants and did not occur).

My previous result had been positive but [my wife’s] had been negative. So we were there for two days, three days before they actually came to us and said “oh, you should be in separate rooms, because now her fourteen days won’t start until you separate rooms… we ended up staying together after that… so, it was a mess

Nicole, April 2020, phase 2, Melbourne, VIC

Following the second wave of infection in Victoria, tests during quarantine became compulsory in some jurisdictions, and were offered in all jurisdictions, with extended quarantine usually required if a person refused testing [17, 18]. Participants in phase 3 reported that they were tested twice, usually very early in the period (such as day 2) and then towards the end (day 10–11). (The testing regimen was different at Howard Springs, with testing upon arrival at the airport and then one test at the facility.) The logic of receiving a negative test result on day 10 but having to stay in hotel quarantine for another 4 days without a further test was queried by participants.

This example highlights a particular risk – not fully appreciated early in the pandemic – that people could be exposed to a higher risk upon entry to or during hotel quarantine due to exposure to others in quarantine, and it may take until day 14 or later for them to return a positive test.

One participant, who was travelling with a partner who had symptoms on arrival, reported being advised by airport medical staff to fake symptoms to enable the couple to stay together.

She’d sort of said, you know, “I don’t want to be separated from my partner.” And [the health official] quickly said, “You better go and give your partner this form, tell him that he’s got some symptoms too*.*

Lloyd, April 2020, phase 1, Sydney, NSW

The couple were then transported to a “health hotel”, where they were separated anyway, as the partner tested positive and the participant remained negative, though both stayed in the health hotel. This participant also reported leaving his room to give vent to feelings of frustration, despite being forbidden to do so under quarantine rules.

There were times that the nurses just didn’t answer the phone. So I’d just sort of open the door, go outside… And I, I went out, and I screamed, you know, and I swore*.*

Lloyd, April 2020, phase 1, Sydney, NSW

This is the only account in our interviews of a participant ‘breaking out’ in hotel quarantine, though participants reported hearing similar outbursts from others, and it was likely only possible because he was housed in a health hotel which did not have security on each floor (at least in phase 1), and he had access to his room key which again was unusual, as most people were locked in without access to a key.

### Risk in non-hotel managed quarantine facilities

Several participants quarantined with children said that alternatives to hotel quarantine should be considered, such as home quarantine with electronic surveillance, or purpose-built facilities. The Howard Springs facility has been held up as an ideal alternative to hotel quarantine, and some features of that location were seen as preferable by the six participants in this study who had stayed there (e.g. access to fresh air). Other aspects of Howard Springs were less well regarded, and there were risks associated with being assigned to that facility rather than a hotel. Howard Springs participants did not describe feeling at risk from COVID-19 infection in quarantine, but did report risks and harms resulting from of the physical environment. The unsuitable design of the facility for families, the heat, and the facility’s location far from non-COVID medical care all contributed to this.

#### Medical care

Three of these six participants had small children, two of whom had accidents while at the facility. One toddler fell from a balcony and cut her head, while another cut his eye on a damaged fitting. Both bled profusely, but the only medical support available was telehealth – not in-person consultation.

It was clear they don’t want to interact with you. Obviously, any person could be a hazard to them. And I asked for some wipes and they wouldn’t send any. So I said, “The only thing I’ve got is Pine-o-Cleen,” and the doctor laughed, which was my point, “or Dettol surface wipes,” and they said, “Oh yeah, the Dettol wipes will be fine. Use that”.

Tina, December 2020, phase 3, Howard Springs, NT

While one of the parents was satisfied that her child’s injury was not serious, the other was very anxious until her son was seen by a doctor after leaving quarantine.

[My toddler] cut his eye. So we immediately called the telehealth… And I was so anxious and scared and crying because I was like, what if something happened to my child’s eye?

Sylvie, January 2021, Howard Springs, NT

The absence of medical care onsite was also a problem for another participant, who was not himself injured but witnessed a fellow detainee in acute pain from a leg injury. Staff at the centre told the participant they could not help, but advised him to call an ambulance himself and attempt to help the man to the facility entrance, disregarding quarantine rules. The ambulance took 4 hours to arrive (Orin, November 2020, phase 3, Howard Springs, NT).

#### Space and amenities

Howard Springs was designed with single able-bodied workers in mind. Accommodation is in separate self-contained rooms with a king single bed, each with its own veranda. This layout was impractical for people quarantining at Howards Springs with children, particularly small ones. Each person in the family was assigned a room, irrespective of age, and there were no adjoining doors or shared family space. This was a problem for families with children too young to be housed separate from parents, in non-adjoining rooms, but with beds too small to comfortably sleep more than one person (cots were available on request). The verandas were not fenced. Participants reported difficulties keeping small children safe and having to buy materials to try to child-proof the space.

While all participants appreciated having outdoor space, the lack of family-friendly features was a significant problem for young families. For people quarantined alone or with older children, however, the facilities offered some advantages, despite it having intermittent Internet access which was a problem for people working remotely. It was also much easier to break rules at Howard Spring as participants weren’t locked in rooms, and participants reported seeing others doing so, wandering ‘illegally’ about the compound.

There was one loveable, nutty woman who broke the rules all the time. She just walked up and down all day long*.*

Tina, December 2020, phase 3, Howard Springs, NT

Some participants found the heat of the Northern Territory difficult to manage, particularly as most of the interviewees in this group had come from a cold winter climate. One reported that she and her daughter had vomited, the result of (self-diagnosed) heatstroke. Many were concerned about the possibility of food poisoning, reporting that food sat in the sun for quite some time during the process of delivery around the facility.

Despite these negative experiences, several participants found the accommodation at Howard Springs to be quite acceptable, and preferable to being ‘locked up’ in a hotel.

It’s very, very humane way of safe-guarding the community health, Australia wide health…putting you into a quarantine facility that feels like it’s actually you know, geared up for quarantining people*.*

Martina, October-November 2020, phase 3, Howard Springs, NT (Martina had been quarantined earlier in Sydney, NSW)

## Discussion

To our knowledge this is the first Australia-wide study focused on the experiences of people in managed quarantine facilities, and we anticipate that our results will contribute to improvements in the continuously evolving quarantine system.

We identified risks both to people in quarantine hotels and to the general public through weak links in the implementation of infection control and broader issues within hotel quarantine. We also discovered concerns with the layout, amenity and systems of medical care at the Howard Springs facility, often held up as an example of a better model of quarantine [19].

When hotel quarantine first commenced and tests were scarce, testing was restricted to individuals who reported symptoms, which some failed to do for various reasons as detailed in the Results. In phase 3, participants were tested at least twice, but the final test was a few days before discharge, with the possibility that they could have been infected but still in the incubation period. Both these factors risked a SARS-CoV-2-positive person being released into the community. This has now been addressed in the Australian system with an extra test scheduled 2 days after discharge from quarantine [20].

Regarding the identified ‘problem’ hotels associated with the second wave of infection in Melbourne, participants reported that one of them had poor hygiene practices in April 2020, with inadequate room cleaning and processes that allowed people with discordant respiratory PCR results to be accommodated together. The issue with ‘filthy’ rooms in this hotel was reported widely by participants quarantined in April, and again by one quarantined 3 months later, suggesting that this was an ongoing problem in the facility. This may be indicative of poor hygiene processes generally that could be related to poor infection control. Alternatively, it could be coincidence, given that since the second wave of infection in Melbourne, SARS CoV-2 infection has leaked from other hotel quarantine facilities that have not be linked with inadequate cleaning. It is not clear whether inadequate security was an issue as participants had different perspectives on this.

Recruitment to this study closed before evidence emerged linking inadequate ventilation in hotel quarantine to transmissions [19]. Nevertheless study participants often felt at risk of acquiring infection enroute to quarantine during administrative processes at the airport, transport to hotels, and within hotel rooms. The requirement to ensure that people experiencing quarantine felt safe is firmly stated in recommendations, from Brooks et al., [21] that officials should ‘take every measure to ensure that this experience is as tolerable as possible for people’ to reduce adverse impacts on mental health, which are common in this setting [22].

With over 20 documented breaches of hotel quarantine in five cities now reported by health authorities, it is clear that there were indeed significant risks [23, 24]. While several ‘leaked’ infections were apparently linked to issues with ventilation linking rooms and corridors [25], at least one was attributed to infection control breaches during transportation [26].

The study also provides some cautions about accommodation at Howard Springs. There have been media representations of the Howard Springs facility as being ‘fit for purpose’ due to its not being in a major city [27] and providing access to outdoor areas for all quarantined people. Our data, however, suggest that for families with small children, multiple single-room units are not ideal and may pose a greater accident risk for small children. In addition, there was need to ensure medical care for non-COVID-19 conditions or accidents.

This study has some limitations. The participants predominantly recruited via Facebook groups about hotel quarantine may have over-represented those with strong views about the experience because they were motivated to join a group. In phase 2, all but one participant was quarantined in one of the two ‘problem’ hotels in Melbourne, so our results from that phase are largely drawn from only one of the two hotels involved in Melbourne’s second wave of infection. The number of participants quarantined at Howard Springs was small. Also, the system is continually being improved and may already have addressed some of the issues identified.

## Conclusions

This study identified systemic issues with infection control in hotel quarantine, which can be further undermined by individual non-compliance. It also showed the need to take better care to reduce risks to personal safety for those in quarantine, both in terms of infection control within hotel quarantine and, in the case of Howard Springs, timely in-person medical care available as needed for non-COVID conditions. Systems of infection control, from the point of leaving the plane to departure from hotel quarantine, need ongoing review to ensure that people entering quarantine are – and recognise themselves to be – protected from known risks of infection at every stage. Medical services at Howard Springs should be examined to ensure that timely and appropriate non-COVID medical services are available in person. Finally, we note that that the accommodation at Howard Springs is unlikely to suit families with small children, who should be accommodated in serviced apartments until purpose-built accommodation is available.

## Data Availability

The datasets generated and/or analysed during the current study are not publicly available because participants were informed that data would be reported in aggregate form only, but are available from the corresponding author on reasonable request.

## Abbreviations

NT: Northern Territory, an Australian jurisdiction
NSW: New South Wales, an Australian state
N-Vivo: Software for managing qualitative research data
PCR: Polymerase chain reaction. This refers to a test to detect genetic material from a specific organism, such as a virus
PPE: Personal protective equipment
QLD: Queensland, an Australian state
SARS-CoV-2: the virus that causes COVID-19-related illness
VIC: Victoria, an Australian state
